# Study on the Spatio–Temporal Evolution of China’s Smart Water Co-Governance in G–E–P Mode

**DOI:** 10.3390/ijerph182312648

**Published:** 2021-11-30

**Authors:** Ning Zhang, Zichen Wang, Hongkai Ru, Haiyang Li

**Affiliations:** 1 School of Management, Hangzhou Dianzi University, Hangzhou 310018, China; hdzhangning@126.com (N.Z.); lihaiyang@hdu.edu.cn (H.L.); 2 School of Economics, Hangzhou Dianzi University, Hangzhou 310018, China; ruhongkai@hdu.edu.cn

**Keywords:** smart water co-governance (SWCG), spatio–temporal evolution, three-stage DEA-Malmquist model, G–E–P mode, efficiency

## Abstract

Smart water co-governance (SWCG) is a fundamental driving force to reduce the water crisis and promote the sustainable development of water resources. To explore the applicability and development of SWCG in different regions, the authors of this paper took 31 provinces of China (with the exception Hong Kong, Macao, and Taiwan) as research districts and used the three-stage data envelopment analysis (DEA) method to measure and compare the efficiency of smart water governance (SWG) in the government–enterprise–public (G–E–P) mode and without public participation in the government–enterprise (G–E) mode in 2019. Then, the Malmquist model was used to measure the spatio–temporal evolution of the G–E–P mode from 2010 to 2019, focusing on the analysis of the top ten provinces of the China Internet Development Index in 2019. According to the empirical analysis, the following results were obtained: (1) the efficiency of SWCG in the G–E–P mode was significantly higher than that in G–E model, as 13 provinces showed a significant decline and 10 provinces had a small change. In addition, SWCG in the G–E–P mode showed a good development trend in the eastern and southern regions. (2) The governance efficiency, pure technical efficiency, and scale efficiency showed upward trends, but the technological progress index and total factor productivity were still low. Therefore, SWG should vigorously promote public participation and the independent implementation of enterprises under the guidance and restriction of the government. Meanwhile, the construction of an SWG infrastructure and the level of science and technology should be strengthened. In addition, each province should adjust the input–output structure according to its redundancy or deficiency, weigh the suitability of the input level and scale, and strengthen the matching and support of the ability of multi-subjects and factors to ensure that an appropriate input–output scale level is reached and the efficiency of SWCG is improved.

## 1. Introduction

The term “water governance” first appeared in 2011 and became a research focus. Lautze (2000) defined it as an open, transparent, fair-participation, efficient, and sustainable governance that aligned water management objectives with local preferences [1]. Water governance is an essential link in the maintenance of water security [2]. Good water governance can effectively reduce the occurrence of the water resources crisis, thus promoting the coordinated and sustainable development of the economy, society, and ecosystem [3]. If the governance issues in water resources, as a global public product, cannot actively be studied, this will impact the development of all parties—especially the study of the water resources’ governance mode, which is needed to help society adapt to the sustainable development governance paradigm [4]. However, in the rapidly developing information age, shared governance and smart governance are inevitable trends of the development of water resource management [5]. Claudia (2019) pointed out that a synergistic hybrid governance could greatly promote the solution to complex water resource management problems in research on the governance mode of a sustainable water governance transformation [6]. Furthermore, in the intelligent era, the development of smart governance is urgent and necessary, especially when focusing on the effective governance process and results obtained with innovative and practical technology [7]. Pang (2021) pointed out that intelligent governance could significantly improve many aspects of the performance of environmental governance, such as strengthening the executive power of the government, increasing public participation, and promoting the green innovation of enterprises [8]. In addition, due to the wide application of intelligent technology in daily life, citizens are increasingly aware of and willing to participate in environmental governance [9]. Therefore, research on smart water co-governance, especially the tradeoff between the effectiveness of scientific and technological decision-making and the fairness of the governance process, is of great significance to the improvement of water resources [10].

Smart water co-governance (SWCG) can be viewed from two aspects: intelligence and collaboration. The concept of collaboration was first put forward by Haken in the field of physics in 1971 and then developed in various fields. In related research on eco-environmental governance, collaborative governance can be understood as a multi-subject governance activity that breaks through a single subject [11]. In addition, sustainable development is at the forefront of international research. Considering the pressure factors of water resources, successful water governance is beneficial for all countries aiming to formulate efficient sustainable development strategies according to their own development conditions, especially water co-governance, which was an effective governance model that gradually increased after government control and subject guidance [12]. The interpretation of the subject of water co-governance is diverse, and can include the government and all kinds of stakeholders, different regions or river basins, or different treatment objects such as sewage, flood and water-saving. No matter how it is classified, the theme of promoting the effective use and sustainable development of water resources will not change [13]. The co-governance of water resources could effectively improve the environment, establish a cooperative relationship between the government and all sectors of society, guide and promote stakeholders’ implementation, and coordinate and alleviate tensions, thus improving governance [14]. With continuous improvements in the social economy and living standards, water management methods are constantly changing from government governance to government-guided governance. However, there was still a gap between water management and public expectations, so it was necessary to promote the public as part of the main body of treatment to improve the efficiency and effectiveness of sewage treatment and ameliorate the water control model [15]. Shi (2012) pointed out that the mode of government guidance, enterprise operation, and public participation was conducive to broadening the development channels of water pollution control and promoting its pluralistic, orderly, and institutionalized development [16]. Simon (2015) pointed out that collaborative governance in the form of stakeholders helped improve the law-abiding nature of society as a whole, which played an essential role in social governance, improving mutual trust, and promoting mutual benefit and win–win situations [17]. Megdal (2017) pointed out that stakeholder participation had an important impact on the strengthening of water resource management and the promotion of the sustainable development of water resources [18]. In a study of urban public crisis governance, Wu (2018) pointed out that promoting public participation was conducive to improving management efficiency, thus promoting the benign interaction of multi-subject collaborative governance [19]. Foster (2021) studied the collaborative governance of water resources in the UK and believed that carrying out close communication and cooperation, improving learning and thinking abilities, and paying attention to the process of collaborative governance (rather than its results) could effectively improve the collaborative governance of water resources [20]. Borowski-Maaser (2021) believed that promoting social learning among stakeholders and defining responsibilities, establishing a knowledge data-sharing platform, and combining the governance structure with governance methods could effectively promote sustainable water resource management. However, there are still many difficulties and challenges in the process and development of collaborative water governance [21]. In addition, in the information age of rapid technological development and efficient use of data, greater attention should be paid to the fairness of shared rights and the openness of information in the process of water co-governance or it would be difficult for it to achieve its true effect and solve complex problems [22]. Zhao (2019) believed that, under the protection of a sound institutional system, the development and application of big data technology would help improve the efficiency of government governance and promote collaborative supervision to improve information sharing and transparency [23]. Grigg (2020) pointed out that intelligent technology could improve the level and ability of governance, promote stakeholders’ participation and sense of responsibility regarding intelligent technology governance, and effectively improve the efficiency of governance [24]. In studying the concept of collaborative governance in smart cities, Nesrine (2021) pointed out that sustainable collaborative networks between governments and external stakeholders could effectively improve the governance environment and performance [25]. He (2021) pointed out that ideal shared urban governance should start from the efficiency of smart governance and the fairness of collaborative governance [26]. On the one hand, information technology and intelligent platforms needed to be promoted; on the other hand, it is necessary to fully coordinate the interests of all parties under the “government + public + enterprise” mode to improve the overall governance level and efficiency [26]. The combination of smart governance and collaborative governance is of great significance when promoting reasonable water resource management. Moreover, the positioning and interest relationships of governance subjects, the research and application of related technologies, and the immediacy and transparency of information sharing are all important for SWCG.

The focus of researches on the achievements and changes of SWCG is the calculation and evolution of governance efficiency. In research exploring governance efficiency and spatio–temporal evolution, most international scholars have chosen the DEA-Malmquist method. However, the efficiency value measured by the traditional DEA method is easily affected by environment factors and random noise, leading to a lack of accuracy; therefore, Fried (1999,2002) introduced the SFA regression analysis to improve the DEA method, thus effectively separating management inefficiency. This was called the three-stage DEA model [27,28]. Subsequently, the model rapidly developed and was applied in various research fields. International scholars have conducted much research on the application of three-stage DEA and its derivative methods in the fields of resource, environment, and ecological governance, including its uses in environmental pollution governance [29,30,31,32], ecological efficiency and governance [33,34,35,36], water resource governance [37,38], the water–energy–food coupling relationship [39,40], energy efficiency and governance [41,42], and agricultural production efficiency [43]. Most scholars have chosen environmental investment or governance costs, employment in related fields or the number of projects under production, environmental protection tax or emissions trading as input variables, environmental governance achievements, energy consumption costs, and the number of treatment facilities as output variables. Research on environmental tax and emissions trading was only conducted in recent years, both of which were carried out through the strong environmental regulation of the government to improve the efficiency of environmental governance under co-governance with the corporate environment, effectively restricting the transfer of pollution. For developing countries, strengthening environmental constraints and regulation could significantly improve environmental behavior [44]. Luo (2019) studied the behavior of the government and enterprises from the perspective of collaborative governance under the environmental tax system and believed that environmental tax was conducive to effectively standardizing the pollutant discharge behavior of enterprises, as well as the structural adjustment of environmental tax can effectively enhance its leverage over environmental governance [45]. Chen (2019) used the DEA model to study the utilization efficiency of water resources in China, pointing out that marketization could effectively improve the utilization rate of water resources, and emphasized the need to pay attention to the flexibility of market water price mechanisms and the rigor of emissions trading [46]. Tian (2020) pointed out that water rights trading helped improve the utilization rate of water resources, thus optimizing the structure of water use and promoting improvements in related technologies [47]. Yu (2021) pointed out that the change from environmental protection fees to taxes promoted the green transformation of heavily polluting enterprises and strengthened the diversified cooperation between the government and enterprises to increase the pressure of enterprise legitimacy and improve efficient government governance [48]. In summation, environmental protection and the energy conservation expenditure of government enterprises, scientific and technological research, development expenditure, the employment of water conservancy and science and technology industries, environmental tax, emission rights, and governance achievements could be chosen as input–output variables in SWCG.

Big data technology has been widely and deeply used in the field of water governance in China, and is constantly developing towards the integration of ideas, the coordination of multiple subjects, and the informationization of technological intelligence with great potential. This technology provides a strong driving force for the enhancement of governance capacity—improving governance efficiency and the governance model [49]. Therefore, it is extremely important to carry out research on related aspects of SWCG, not only to test the current development and implementation status to find the defects and stop them in time for improvements to be made but also to promote the sustainable and coordinated development of human society and the ecological environment. Based on the collaborative governance perspective, the authors of this paper used the three-stage DEA-Malmquist method to measure and study the efficiency and spatio–temporal evolution of China’s SWCG under the G–E–P model. The innovation of this paper is that, as a new concept, quantitative research into SWCG is still insufficient. The authors of this paper used the public factors as environment variables in calculations to compare and analyze the results of SWCG under the G–E–P and G–E modes. The paper’s structure is as follows: Section 2 presents the research design, including index setting and research methods. Section 3 presents the empirical analysis, including data sources, three-stage DEA result analysis, and Malmquist index measurement of spatio–temporal evolution. Section 4 is the discussion; and Section 5 is the conclusion. The appendix contains the complete data on the results of the text presentation data.

## 2. Research Design

### 2.1. Index Setting

Chang (2021) took asset investment and labor as input variables in a study of wastewater treatment efficiency, and they pointed out that the increasing investment in environmental treatment has had an obvious effect on improvements in water treatment [50]. Long (2020), in the study of the efficiency of green governance in the Yangtze River Economic Belt, pointed out that government support for technological innovation will help promote the level of national green governance, thus promoting sustainable regional development [51]. Liu (2020) also pointed out that increasing government expenditure on science and technology can effectively improve the efficiency of resource and environmental governance [33]. In addition, Li (2021) believed that expanding and improving the government’s financial investment in the field of environmental protection would help to guide the entry of social capital, thus expanding the volume of capital, promoting the diversification of governance subjects, and providing an effective guarantee for improved results regarding environmental governance [52]. Liu (2014) believed that the construction of an urban water supply and drainage network was a key point in measuring the level of sewage treatment, especially after the combination of data collection and information functions. Therefore, it would be convenient for the government, enterprises, and the public to obtain real-time and accurate information [53]. Bi (2011) pointed out that the discharge permit system was an effective measure to control water pollution in China, as it could promote the efficiency of water pollution control and the reliability of results [54]. Zhou (2020) believed that improving the government’s environmental regulation and supervision could improve the regional ecological level; for example, increasing environmental taxes would effectively regulate the pollution behavior of enterprises [36]. Li (2019) also regarded environmental tax as a means of controlling corporate pollution emissions and making up for the external environmental losses caused by discharge pollution, stimulating enterprise initiatives regarding environmental protection, and promoting their green transformation, which is conducive to the protection of water resources and the environment [55]. Hu (2020) studied the utilization rate of water resources and the effect of sewage treatment on rural water pollution and pointed out that reducing the water consumption for economic development and improving the capacity of water pollution treatment could promote the treatment and remediation of rural water pollution [56].

According to the literature review in the introduction and the views of the above-mentioned scholars, the authors of this paper designed and selected input–output variables and environment variables for SWCG, with the public’ impact on collaborative governance used as environment variables. Thus, we were able to compare the difference in the SWCG’s efficiency before and after excluding the public. The specific index system of input–output variables is shown in Table 1.

Descriptive statistics of each indexes are shown in Table 2.

### 2.2. Research Methodology

Data envelopment analysis (DEA) is a flexible, simple, effective, and objective non-parametric method based on the relative efficiency of evaluated objects. It has been widely accepted and adopted by scholars in the fields of resources and environment, farmland water conservancy, and health care. However, the efficiency value, measured by traditional DEA, is not directly caused by management inefficiency; therefore, Fried (1999) successively introduced environment factors and random noise into the DEA model and modified the process with the SFA method to improve its limitation. Considering that DEA can only horizontally compare the efficiency of SWCG, the Malmquist index model was introduced, and then the efficiency was longitudinally and dynamically analyzed. Therefore, the authors of this paper used the three-stage DEA-Malmquist method to calculate SWCG efficiency.

**Figure 1:**
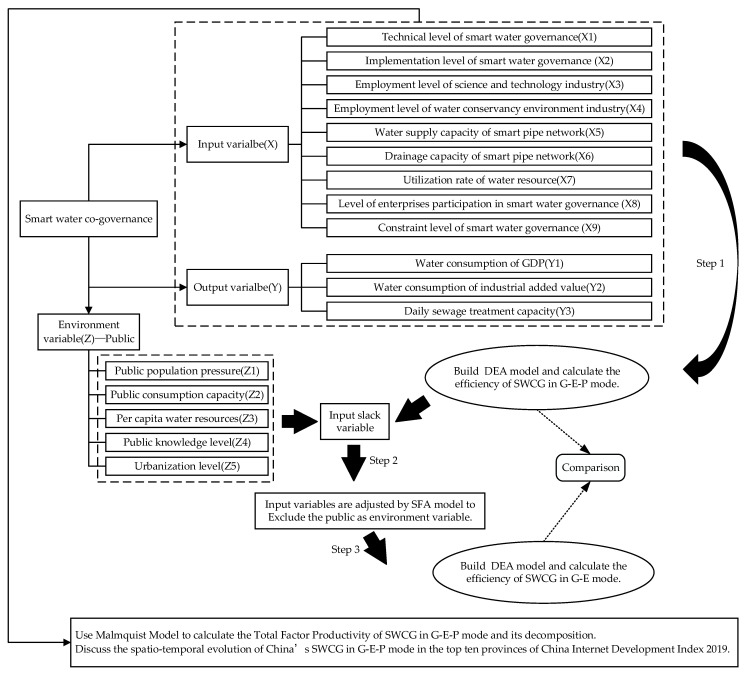
Flow chart of three-stage DEA-Malmquist model for SWCG.

Specifically, first, the traditional DEA model was used to initially evaluate the efficiency of SWCG among the government, enterprises, and the public; secondly, SFA was used to avoid statistical noise and re-evaluate the efficiency of SWCG between the government and enterprises after excluding the public as an environmental factor; finally, the Malmquist model was used to calculate the dynamic changes in SWCG efficiency. The specific flow chart is shown in Figure 1.

#### 2.2.1. First Stage DEA

In the first stage, the original input–output data were used to evaluate the initial efficiency. DEA includes two non-radial models—CCR and BCC. The CCR model is usually suitable for the circumstance of constant returns to scale (RTS), while the BCC model is based on the CCR model with variable RTS and can obtain a pure technical efficiency, with the exception of scale efficiency [57]. Therefore, for the first and third stages of this paper, the BCC model was used to calculate technical efficiency, pure technical efficiency, and scale efficiency. The equation is as follows:

(1) $$\begin{array}{l} \min[\theta -\varepsilon (\sum_{i=1}^{m}S_{i}^{-}+\sum_{r=1}^{s}S_{r}^{+})]\\ s.t.\left\{ \begin{array}{l} \sum_{j=1}^{n}\lambda_{j}X_{j}+S_{i}^{-}=\theta X_{0}\\ \sum_{j=1}^{n}\lambda_{j}Y_{j}-S_{r}^{+}=Y_{0}\\ \sum_{j=1}^{n}\lambda_{j}=1\\ \theta ,\lambda_{j},S_{i}^{-},S_{r}^{+}\geq 0 \end{array}\right. \end{array}$$

*j* = 1, 2, …, *n* is the decision-making unit; *θ* is the efficiency value of the decision-making unit; *ε* represents the infinitesimal quantity of non-Archimedes infinitesimal quantity, which can effectively judge the effectiveness of relative efficiency, and its general value is 10^−6^; *S_i_^−^* and *S_r_^+^* are the remaining variable and slack variables, respectively; and *X_j_* and *Y_j_* are the input and output variables of the j decision-making unit, respectively, $\overset{n}{\underset{j=1}{\sum }}X_{j}\;{=X}_{0},\;\overset{n}{\underset{j=1}{\sum }}Y_{j}{=Y}_{0}$. When *θ* = 1 and *S_i_^−^* = *S_r_^+^ =* 0, the decision-making unit is strongly DEA efficient; when *θ* = 1 and *S_i_^−^* > 0, *S_r_^+^ >* 0, the decision-making unit is weakly DEA efficient; when *θ* < 1, the decision-making unit is not DEA efficient.

#### 2.2.2. Second Stage SFA

The second stage mainly focuses on relaxation variables, which Fried believes can reflect the initial inefficiency, which is composed of environmental factors, management inefficiency, and statistical noise. Therefore, with the help of SFA regression, the first stage of relaxation variables is decomposed into the abovementioned three effects, and the environmental variables and mixed error terms of the first stage relaxation variables are regressed. In this paper, the quasi-SFA regression equation was established as follows:

(2) $$S_{nm}=f^{n}(Z_{m};\beta_{n})+\nu_{nm}+\mu_{nm};n=1,2,\cdots ,I;m=1,2,\cdots ,N$$

*n* = 1, 2, …, *I*; *m* = 1, 2, …, *N*; *S_nm_* is the relaxation variable of the nth input of the m decision-making unit; *Z_m_* = (*Z_*1*m_*_,_ *Z_*2*m_*, …, *Z_pm_*) is the *p* environment variables; *β_n_* is the coefficient of environmental variables; *f^n^*(*Z_m_*; *β_n_*) is the mapping of environmental variables to relaxation variables; and *v_nm_* + *μ_nm_* is a mixed error term, where *v_nm_* is a random disturbance term and *μ_nm_* is a management inefficiency—both are independent and not related. The adjustment equation of the input variable is as follows:

(3) $$X_{nm}^{*}=X_{nm}+[\max(f(Z_{m};{\overset{\wedge }{\beta }}_{n}))-f(Z_{m};{\overset{\wedge }{\beta }}_{n})]+[\max(\nu_{nm})-\nu_{nm}]\;$$

*X_nm_* and X^*^_nm_ are input variables before and after adjustment, respectively; $\max(f(Z_{m};{\overset{\wedge }{\beta }}_{n}))-f(Z_{m};{\overset{\wedge }{\beta }}_{n})$ is an adjustment to environmental factors; and ${\max(v}_{nm}{)\;-\;v}_{nm}$ is an adjustment to random disturbances. The former aims to put all DMUs in the worst environment, and the latter aims to put all DMUs in the same natural state.

The equation for separating inefficient managing items is as follows:

(4) $$E\left(\mu |\varepsilon \right)=\sigma^{{}_{*}}\left[\frac{\phi (\lambda \frac{\varepsilon }{\sigma })}{\Phi (\frac{\lambda \varepsilon }{\sigma })}+\frac{\lambda \varepsilon }{\sigma }\right]$$

where $\sigma =\sqrt{\sigma_{\mu }{}^{2}+\sigma_{\nu }{}^{2}},\sigma^{{}_{*}}=\left(\sigma_{\mu }\sigma_{\nu }\right)/\sigma ,\lambda =\sigma_{\mu }/\sigma_{\nu }$.

The equation for calculating the random error term *v* is as follows:

(5) $$E\left[\nu_{nm}|\nu_{nm}+\mu_{nm}\right]=S_{nm}-f\left(Z_{m};\beta_{n}\right)-E\left[\mu_{nm}|\nu_{nm}+\mu_{nm}\right]$$

#### 2.2.3. Third Stage DEA

Using the adjusted input variable from Equation (1), the efficiency of each decision-making unit was re-calculated. That is, after excluding the public factors, the cooperative governance efficiency of the government and enterprises was measured.

#### 2.2.4. Malmquist Model

The Malmquist index was first proposed by Malmquist in 1953 and began to be widely used to measure the change in production efficiency after Fare et al. combined it with the DEA method in 1994. The exponential decomposition equation is as follows:

(6) $$TFPCH=\left\{ \begin{array}{l} EFFCH\times TECHCH=PECH\times SECH\times TECHCH\\ \frac{D_{t}^{u}(x_{t},y_{t})}{D_{t-1}^{u}(x_{t-1},y_{t-1})}\times \left[\frac{\frac{D_{t}^{C}(x_{t},y_{t})}{D_{t-1}^{C}(x_{t-1},y_{t-1})}}{\frac{D_{t}^{u}(x_{t},y_{t})}{D_{t-1}^{u}(x_{t-1},y_{t-1})}}\right]\times {\left[\frac{D_{t-1}^{C}(x_{t-1},y_{t-1})}{D_{t}^{C}(x_{t-1},y_{t-1})}\times \frac{D_{t-1}^{C}(x_{t},y_{t})}{D_{t}^{C}(x_{t},y_{t})}\right]}^{\frac{1}{2}} \end{array}\right.$$

Among them:

*TFPCH* represents total factor productivity;

$PECH=D_{t}^{u}(x_{t},y_{t})/D_{t-1}^{u}(x_{t-1},y_{t-1})$ represents pure technical efficiency change index;

$SECH=\left[D_{t}^{C}(x_{t},y_{t}\right)/D_{t-1}^{C}(x_{t-1},y_{t-1})]/\left[D_{t}^{u}(x_{t},y_{t})/D_{t-1}^{u}(x_{t-1},y_{t-1})\right]$ represents the scale efficiency change index;

EFFCH=PECH×SECH represents the technical efficiency change index; and

$TECHCH={\left[\left(D_{t-1}^{C}(x_{t-1},y_{t-1})/D_{t}^{C}(x_{t-1},y_{t-1})\right)\times \left(D_{t-1}^{C}(x_{t},y_{t})/D_{t}^{C}(x_{t},y_{t})\right)\right]}^{\frac{1}{2}}$ represents the technological progress index.

## 3. Empirical Analysis

### 3.1. Data Source

The authors of this paper selected the input, output, and environmental index data of SWCG in various provinces from 2010 to 2019, mainly from the China Statistical Yearbook from 2010 to 2019. The authors of this paper made a horizontal comparison of the situation among provinces in recent years with 2019 data and analyzed the dynamic changes in the data from 2010 to 2019. Moreover, to better study the differences between different provinces at the level of internet development, according to the “Blue Book of China Internet Development report 2020” (a book that aims to record the history of China’s internet development, innovative achievements, existing problems, and a summary of the current situation and experience, which includes modules such as information infrastructure construction, information technology development, digital economy development, e-government development, network content and security construction), the authors of this paper compared the top ten provinces of the Internet Development Index (which covers a comprehensive evaluation of China’s information infrastructure construction, innovation capability, digital economy development, internet application, network security and governance, as well as providing a quantitative reference basis for the development of the internet in the whole country and provinces) in 2019 with other provinces to further analyze the results, as shown in Table 3.

### 3.2. Comparison of the First and Third Stage

**Figure 2:**
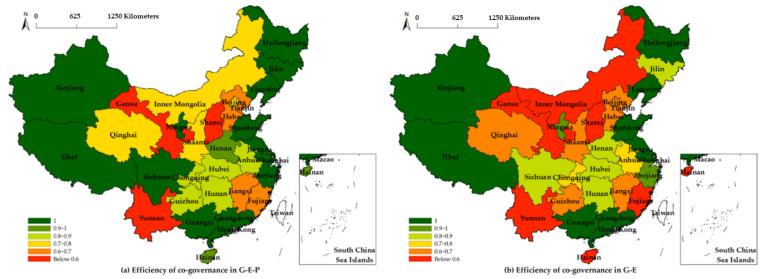
The efficiency of SWCG with different entities.

**Figure A1:**
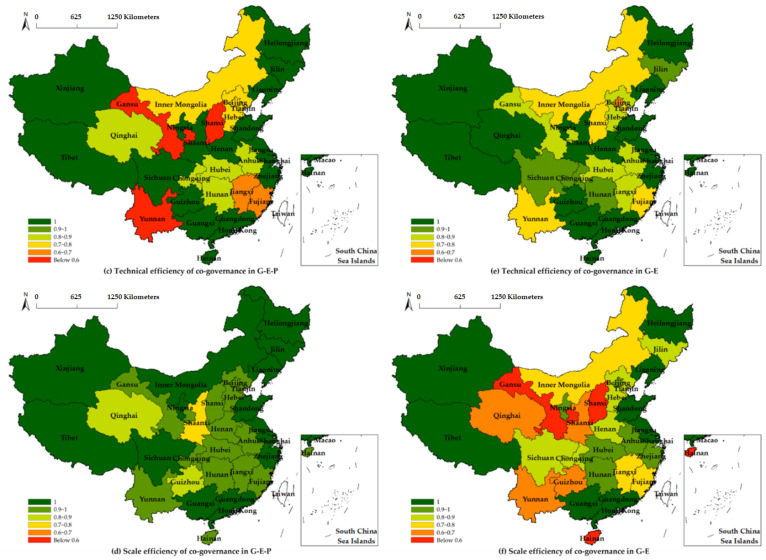
The technical and scale efficiency of SWCG with different entities.

According to the variable index system in Table 1, the input and output indicators were selected and combined with Equation (1). The efficiency values of 31 provinces in China (excluding Hong Kong, Macao, and Taiwan) were calculated using the DEAP2.1 software. The governance efficiency results of the first and third stages are shown in Figure 2, the specific calculation results are shown in Table A1, and the technical and scale efficiency are shown in Figure A1.

It can be seen from Figure 2 that the efficiency of SWCG in the G–E–P mode is generally better than that in the G–E mode. Among them, 13 provinces showed a significant decline in efficiency (that is, the color shifted to a lower level), 8 provinces maintained an efficiency of 1, and 10 provinces remained at the same level. Combined with Table A1, it can be seen that, except for Jiangxi, Hubei, and the 8 provinces with an efficiency of 1, all other provinces experienced a decline in efficiency. Among them, the co-governance efficiency of G–E–P in Jilin, Zhejiang, Ningxia, and Sichuan provinces decreased to a lower level, while Inner Mongolia, Sichuan, Guizhou, and Hainan showed a great-leap-forward decline. In terms of scale returns, 14, 11, and 6 provinces showed constant, increasing, and decreasing scale returns, respectively, under the G–E–P model, and there was an overall increasing trend, except for 9 provinces with the same scale returns, under the G–E model. It can be seen that, after excluding the public, the efficiency of collaborative governance tended to notably decline. Therefore, regarding SWCG, as a new means of water control, shows that it is not enough to rely on government policies to drive enterprise behavior; public participation is also needed to improve the level of SWCG, improve ecological resources and environment, and promote the sustainable development of the economy, society, ecological environment, and water resources.

In addition, as can be seen from Figure 2, regardless of the collaborative governance model, the results of the overall efficiency of SWCG in China show that the eastern part is better than the central and western parts, and the south is better than the north. First of all, the eastern region has always been a leading state in terms of domestic economic development. Therefore, its investment in water resource protection and utilization is better than that of the central and western regions. With the help of the superior level of scientific research and development, the eastern region has a higher level of intelligent water control development, thus showing a higher efficiency. Secondly, Eastern China is a coastal area, and its precipitation resources are more abundant than those in the central and western regions, so it has advantages in terms of total water resources and climate. The “South-to-North Water transfer” water conservancy project reflects the general distribution and water resource problems in China. Finally, it has been found that the efficiency of SWCG in Xinjiang and Tibet has always been 1 due to the low level of science and technology, which is because the two provinces have a vast territory, a small population, and a low level of industrialization. Moreover, due to the abundant content of water resources due to topography, the degree of utilization and destruction of water resources is relatively low, so a high level of governance efficiency can be maintained under weak scientific and technological levels.

From the comparison between a and b in Figure A1, it can be seen that the efficiency of G–E–P SWCG is mainly affected by low technical efficiency, especially in the central and western regions, as well as in Yunnan and other provinces. After the joint participation of the government, enterprises, and the public, the scale efficiency was found to show an overall high level, which was lower than 0.8 in Shaanxi alone. However, at this time, the technical efficiency is not enough to support the high level of participation. Therefore, all provinces need to continue to improve the technical level of SWCG, especially in the central and western regions. From the comparison between c and d in Figure A1, it can be seen that the efficiency of SWCG in G–E is mainly affected by low scale efficiency, as only eight provinces were found to have a scale efficiency of 1, and the technical efficiency was relatively improved after the scale reduction.

Whether in terms of the SWCG efficiency or scale efficiency, only the efficiency of government and enterprise participation in governance was observed to show a downward trend after the lack of public participation. Therefore, SWCG requires the joint efforts of many subjects, and it is not enough to rely on the unilateral promotion of the government, policies, and rules to restrict the behavior of enterprises; enterprises and the public also need to improve their water-use behavior and save water by themselves. As a driving force, the government should improve the technical level of SWCG and work together to achieve the goal of the sustainable development of resources, environment, and human society.

### 3.3. Analysis of the Second Stage

To study the different results for SWCG efficiency between G–E–P and G–E, the public was excluded as an environment variable in the process of the SFA model. According to Table 1 and Equations (2)–(5), after obtaining the mixed error term, Frontier4.1 was used to calculate the relationship between input variables and environment variables and the original input was adjusted; the adjustment process of input variables is not shown due to layout reasons. The results of the SFA analysis are shown in Table 4, and the results of mixed error terms are shown in Table A2.

As can be seen from Table 4, the environment variables passed the *t*-value test for the input slack variables of smart water control technology, implementation, technical employment, enterprise participation, and restraint level, which shows that environment variables have a significant impact on green governance investment redundancy. However, they were found to have no significant impact on the professional employment level of the water conservancy environment, the water supply and drainage capacity of intelligent pipe networks, and the utilization rate of water resources. Secondly, the threshold value in all regression models was close to 1, indicating that the public, as an environmental factor, dominates the cooperative management efficiency of SWCG and the impact of random errors is not significant; finally, all the input variables passed the *LR* likelihood ratio test, indicating invalid rate items.

Among the non-significant indicators, the water supply and drainage capacity of the smart pipe network and the utilization rate of water resources, the planning and construction of the water supply and drainage pipe network, and the exploitation and utilization of water resources were found to be mainly determined by the government and cooperation with enterprises. Therefore, the chance of direct public participation is not high, which has an insignificant impact. However, the public’s insignificant impact on the employment level of water conservancy and environmental professionals is difficult to explain here, and new research is needed for an in-depth discussion.

### 3.4. Analysis of Malmquist Results

As the three-stage DEA only showed the horizontal static efficiency of G–E–P SWCG, to further analyze the dynamic trend in different years, the authors of this paper used the original data (because the data on enterprises participating at the SWG level are missing and therefore deleted in the Malmquist analysis). The Malmquist index method was used to measure the dynamic changes in the efficiency of G–E–P SWCG from 2010 to 2019, and the top ten provinces of the Internet Development Index were selected to carry out a further horizontal analysis. The dynamic efficiency results of 31 provinces are shown in Table A3 and Table A4.

#### 3.4.1. 2010–2019 Total Evolution Results

**Figure 3:**
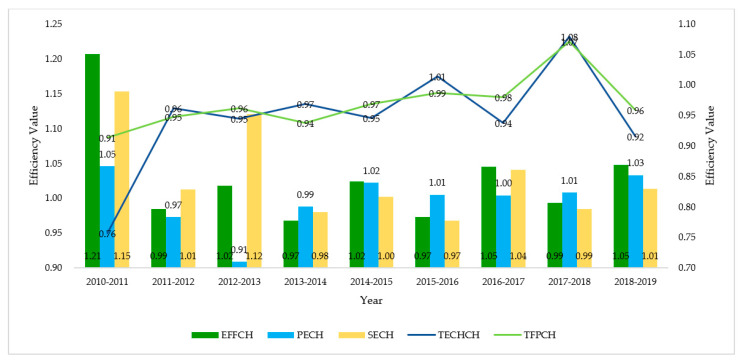
Malmquist results of SWCG evolution in 2010–2019.

According to Equation (6), the panel data from 2010 to 2019 were selected, and the Malmquist index was calculated using DEAP2.1 software [30]. Due to the large calculation results, the authors of this paper intercepted the average value of 31 provinces’ SWCG efficiency as the overall dynamic efficiency change result, and the specific results are shown in Figure 3.

As can be seen from Figure 3, the overall efficiency, pure technical efficiency, and scale efficiency of G–E–P SWCG showed an upward trend from 2010 to 2019, with only slight declines over a few years where the pure technical efficiency decreased by nearly 0.1 from 2012 to 2013, indicating that the technical level of that year was not enough to support the expansion of scale. The technical progress index was only greater than 1 in 2015–2016 and 2017–2018; the results of the other years were all less than 1, and the index for 2010–2011 was only 0.76, indicating that the technical level of SWCG was low at first, and the overall progress in the past decade was still not satisfactory. Therefore, the country still needs to increase its efforts to promote the development of industries related to SWCG and improvements at the technical level. The total factor productivity was only more than 1 from 2017 to 2018, and it decreased slightly in other years, indicating that the input and output of SWCG did not reach a high matching level and were mainly limited by the low technical level. Generally speaking, the overall efficiency level of G–E–P SWCG in 2010–2019 fluctuated little but showed a rising trend. Therefore, as a new concept and route, SWCG must require all parties to increase their participation and input to improve governance efficiency.

#### 3.4.2. Top Ten Provinces in China Internet Development Index

**Figure 4:**
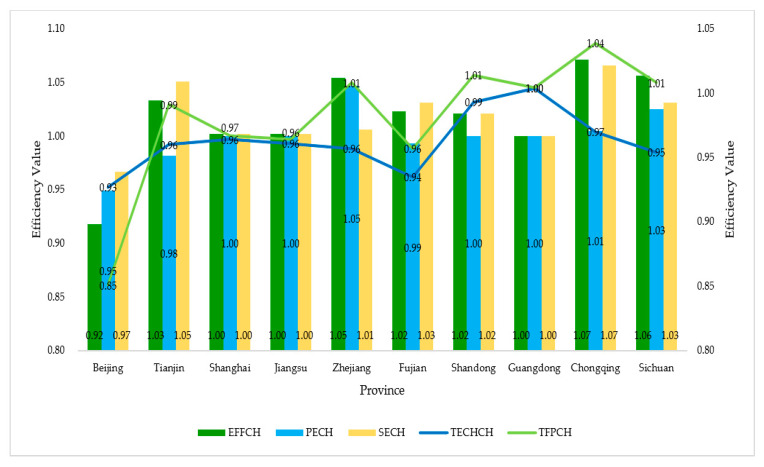
Malmquist results of top ten provinces in the 2019 China Internet Development Index.

To further compare the changes in the efficiency of SWCG in provinces with a higher level of internet development, the authors of this paper selected the top ten provinces in the 2019 China Internet Development Index as the research object, calculated it, and selected the mean as the evaluation result. The specific results are shown in Figure 4.

As can be seen from Figure 4, in the top ten provinces in China’s Internet Development Index, although the level of science and technology was found to have a leading position in the country, the efficiency of G–E–P SWCG did not reach a good level. Only Zhejiang and Sichuan reached the national average in terms of comprehensive efficiency, pure technical efficiency, and scale efficiency. In addition, Chongqing reached the average for comprehensive efficiency and scale efficiency. Tianjin, Fujian, and Shandong only exceeded the national average for scale efficiency. Beijing’s efficiency was less than 1, indicating that the construction, application, and promotion of the concept of SWCG in Beijing is not perfect, and there is also a lack of technological research and development and application in this area. Shanghai, Jiangsu, and Guangdong did not have a comprehensive efficiency, pure technical efficiency, and scale efficiency of 1, and they did not reach the national average level, indicating that the three places’ knowledge of water control is still in a relatively backward stage. Good use is not being made of scientific and technological abilities, and there is no effort to promote the willingness of all parties to participate. In Zhejiang, since the plan of “five-water co-governance” was put forward in 2014, the province has reached a high level in terms of scientific and technological research, development investment, workforce investment, publicity efforts, and citizen participation in water resource governance, thus promoting the development of SWCG in Zhejiang in all directions due to an outstanding efficiency of collaborative governance.

For the technical progress index, although all provinces exceed the national average, only Guangdong was found to reach 1. Total productivity reached average levels in all provinces, except Beijing, but only Zhejiang, Shandong, Guangdong, Chongqing, and Sichuan improved. Therefore, it can be seen that the technical level limits growth in total factor productivity. Although the SWCG scale has been expanded, the overall technical level is too low due to the low technical level impacting total factor productivity.

Generally speaking, the construction of intelligent water control should focus on the improvement and application of the relevant technological level, and the increase in scale efficiency shows that the participation of all parties has neem very strong. It is precise because of this that it is necessary to continuously improve the technical capacity of the government in the implementation of supervision and governance, the utilization and discharge of water resources by enterprises, and the public’s daily water use and online participation to improve the efficiency and level of overall collaborative governance.

## 4. Discussion

According to the above analysis, the SWCG in China is still at a low level in the current stage. The technical level, in particular, still needs to increase investments to improve pure technical efficiency. In recent years, with the vigorous development and promotion of the concepts of national ecological protection, resource development, and recycling, as well as the progress in people’s living standards and ideas (whether using SWG or other resources) and environmental protection activities, the willingness of the public and enterprises to participate is unprecedentedly high. In daily life, the public more effectively utilizes daily water resources through water-saving and other behaviors. For polluting enterprises, stealing discharge behavior has been greatly reduced compared to previous years, and the investment in sewage source treatment has increased. With policy support and the help of the government, the level of sewage discharge has been significantly reduced. The government has opened up a variety of water resource governance models and approaches, including online platform construction, paid emissions trading, environmental tax restrictions, and the introduction of several policies and regulations. On the one hand, non-standard behavior is restricted with tough conditions, but on the other hand, multi-parties are encouraged to participate in the construction of water resource protection and promote the sustainable development of ecological resources and environment.

To effectively discover the problems existing in non-DEA efficient provinces, the authors of this paper collated the input redundancy rate and output deficiency rate, as shown in Table 5.

As can be seen from Table 5, the utilization rate of water resources was found to be the highest, with an average redundancy rate of 76%. The implementation of SWG and the level of constraints were relatively surplus, with average redundancy rates of 48% and 53%, respectively. The average redundancy rate of other input variables was at a low level; the deficiency rate of output variables was all 20%, which shows that the overall output was still considerable.

Most of the lack of output was found to be concentrated in the central and western regions, and there was a phenomenon of insufficient input. Therefore, it is necessary to increase the investment in SWG in the central and western regions, especially by reducing the utilization rate of water resources and integrating government environmental protection investment and environmental tax settings, which can effectively increase the level of output. In addition, although Liaoning did not show a deficiency in terms of output, its input was found to be redundant, which shows that if DEA is effective and its resource allocation is unreasonable, with an excessive input needed to obtain an effective output. Therefore, Liaoning should focus on improving the input collocation structure to effectively improve the input resource utilization rate.

As far as the utilization rate of water resources is concerned, there was a serious input redundancy of up to 1356% in Tianjin, indicating that the utilization of water resources in Tianjin is extremely unreasonable. Yao (2021) also pointed out that the utilization efficiency of comprehensive water resources in Tianjin is not high and has shown a downward trend [58]. Therefore, Tianjin needs to focus on improving the water-use structure of various industries, improving sewage treatment capacity and the level of water recycling. At the same time, it also needs to optimize the arrangement of underground pipe networks to improve the unreasonable use of water resources. Regarding the implementation level of SWG, many provinces have an excessive investment in environmental protection, indicating an unreasonable allocation of funds in resources and the environment. Therefore, each province should effectively plan their founding in terms of investments in environmental protection budgets to effectively increase the use of environmental protection funds. Regarding the restriction level of intelligent water control, the predecessor of the environmental tax is the sewage charge, which is better for planning environmental protection behaviors. Yu (2021) pointed out that an environmental tax can effectively improve the green transformation potential of enterprises and the compulsion and rigor of law enforcement, thus reducing the cost of government restraint and control; however, if the range of control is too large, it will weaken the promotion of the green transformation of enterprises [48]. Therefore, areas where the investment of environmental tax is too redundant should improve the implementation details, ensure effective implementation, and improve the pressure of environmental legitimacy of enterprises. In addition, the government should guide enterprises to improve their water-use behavior and jointly promote the green transformation of enterprises through tax relief and other policies to promote the sustainable development of the economy and society, natural resources, and ecological environment.

In addition, the Henan, Hainan, Guizhou, and Shaanxi provinces were found to have no input redundancy and an insufficient output, showing non-DEA-efficiency, indicating that pure technical efficiency is at a high level but the scale is still lacking. Therefore, the four provinces were found to have strengthened the publicity of SWG, promoted multi-party participation to expand the effectively overall scale, and should constantly improve the technical level to promote the efficiency of SWCG.

Generally speaking, the promotion of SWCG requires the matching and support of multi-subjects and factors, and a rapid expansion of scale in a short period is not enough to solve the practical problem of low governance efficiency. It may even cause problems, such as a lack of technological progress and imbalance in input matching, resulting in redundancy or the insufficient output of some input variables. Therefore, each province should adjust its input–output structure according to its redundancy or deficiency to ensure that the input–output scale reaches an appropriate level.

## 5. Conclusions

In this paper, the three-stage DEA model was used to compare and analyze the efficiency of SWCG in 31 provinces of China in 2019 under different participant modes, and the Malmquist index model was used to dynamically analyze the efficiency of SWCG under the G–E–P mode from 2010 to 2019. The conclusions are as follows:

1. The efficiency of SWCG in G–E–P mode was significantly higher than that in the G–E mode. On the whole, the eastern part was better than the central and western regions, and the south was better than the north, in which 13 provinces showed an obvious decline inefficiency, the efficiency of 8 provinces was 1, and the efficiency of 10 provinces varied in a small range. After excluding the public, although the technical efficiency improved, the scale efficiency significantly decreased, resulting in a significant decline in overall efficiency.
2. From 2010 to 2019, the efficiency, pure technical efficiency, and scale efficiency of SWCG in the G–E–P mode showed an overall upward trend, and the technological progress index and total factor productivity fluctuated little but were still at a low level. In addition, although the top ten provinces of the China Internet Development Index are in a leading position in the country at the scientific and technical levels, the efficiency of SWCG in the G–E–P mode did not show a good level; only Zhejiang and Sichuan exceeded the mean level, while Beijing lagged.
3. In the G–E–P mode, there were 13 DEA-efficiency provinces for SWCG in 31 provinces, among which Liaoning had a weak DEA-efficiency with input redundancy. Among the non-DEA-efficiency provinces, Henan, Hainan, Guizhou, and Shaanxi had no input redundancy and output deficiency. The utilization rate of water resources was the highest overall, with an average redundancy rate of 76%. The implementation of SWG and the level of constraints are relatively surplus, with an average redundancy rate of 48% and 53%, respectively, and the average redundancy rate of other input variables is low.

According to the above conclusions, this paper puts forward some policy suggestions:

1. In the process of promoting SWCG under the G–E–P mode, we should insist on taking the government as the lead and giving full play to the supervisory role of public participation under the guidance and restraint of the government and the independent implementation of enterprises. In addition, it is necessary to promote public participation in SWCG as the starting point, to improve the local water environment, provide the public with a good living environment, and enhance people’s well-being. Additionally, it is important to emphasize the ideological importance of public participation in SWCG, and to promote, encourage and guide the public to actively participate in SWCG. A situation can then be formed under the integration of social capital and efficient management of public participation in water governance, in which the government, enterprises, and the public discuss, build and share ideas.
2. The construction of an information platform for water resources protection needs to be widely promoted. Information sharing is the basis of synergetic governance and an important condition for effective water resource governance. The open sharing of information among government, enterprises and the public can give the public a sense of identity in water resource management, and enhance the public’s sense of responsibility, attention and participation in SWCG. Therefore, we should strengthen the information communication between government, enterprises and the public, unblock the channels of social supervision and public opinion supervision, and promote the construction of an information sharing platform to ensure the timely dissemination of information.
3. The government should conscientiously implement the policy of independent innovation, strengthen diversified investment in the development of SWCG technology innovation, co-ordinate scientific and technological resources, encourage and attract R&D personnel, promote improvements in water control technology innovation ability, attach importance to the role of scientific and technological innovation talent development in promoting the level of SWCG technology, vigorously train a new batch of high-level, intelligent water control talents, and fundamentally solve the “being hit in the throat” technical problems. We should actively promote the innovation and R&D investment of related industries, guide social capital towards the water control industry, participate in the R&D and promotion of SWCG products and technologies, and build a water control system of “public participation, technological iteration and innovation” to promote the optimal development of SWCG.
4. The government should objectively evaluate and analyze the efficiency of the self-examination of water resource utilization, actively adjust the input–output institutions and mechanisms according to the objective situation of redundancy or insufficiency, weigh the suitability of the water control input scale and water control scale, and explore the balance point of input–output and water resource utilization rates to achieve the best matching. With the best balance point, we should comprehensively consider and formulate corresponding policies and measures, strengthen the matching and supporting ability of multiple subjects and elements with diversified investment, reduce the redundancy rate, fill the shortage and improve the utilization rate of water resources, so that the scale of input and output is suitable for the actual situation of each province, to achieve the SWCG goal with a low redundancy rate, no investment shortage and a high utilization rate of water resources.
5. Establish and improve the “delegation-agent” mechanism of interaction between central and local governments, and give full play to the policy planning and leadership role of the central government. It should encourage the devolution of some powers to local governments and clarify the interests and responsibilities of each party as well as the objectives of water resource governance planning. Strengthen the communication and coordination between the central and local governments in the area of SWCG, so as to effectively prevent and solve the problem of information asymmetry in the process of communication between the upper and lower levels.

In addition, there are still some limitations in this paper. Smart water governance is a new research object and future development trend, and collaborative governance is an effective cooperative governance model. Therefore, it is of great practical significance to study the combination of smart water co-governance. However, although this is the innovation of this paper, it is also a deficiency. The current SWCG theory is not perfect and does not necessarily perfectly match the governance model; for example, the authors of this paper did not use the non-expected output and took the negative indicators as input indicators when calculating, so there might have been defects in the selection and construction of the index system. As a result, there was a certain deviation in the evaluation results. Secondly, this model is more suitable for the calculation of efficiency, but it is difficult to obtain the actual impact of specific indicators on final results. In the Malmquist index analysis, incomplete data collection was found to lead to a lack of enterprise participation in SWCG, which may have impacted the final results. Finally, due to the particularity of China’s regional development, some conclusions in this paper could be explained by combining the specific conditions of the region, which has led to some subjective characteristics in some conclusions. The authors of this paper did not further explore and analyze the specific impact of input variables on output, nor did they provide weights according to the regional situation when calculating the overall efficiency; all these can be used as ideas to further improve the research in this area.

## Figures and Tables

**Table 1:** Input, output, and environment variables of SWCG index system.

Index Type	Index	Index Interpretation
Input variable (X)	Technical level of SWG (X1)	Take science and technology financial expenditure as the government’s technical level of SWG.
	Implementation level of SWG (X2)	Take energy-saving and environmental protection expenditures as the government’s implementation level of SWG.
	The employment level of the science and technology (X3)	Take the number of employees in scientific research and technology as the employment level of the science and technology industry.
	The employment level of the water conservancy environment (X4)	Take the number of employees in the water conservancy environment as the employment level of the water conservancy environment industry.
	The water supply capacity of the smart pipe network (X5)	Take the density of water supply pipelines as the water supply capacity of the smart pipe network.
	Drainage capacity of smart pipe network (X6)	Take the density of drainage pipes as the drainage capacity of the smart pipe network.
	The utilization rate of water resources (X7)	Take the utilization rate of water resources as the level of water resource development and utilization.
	Level of enterprises participation in SWG (X8)	Take the number of pollutant discharge permits as the level of enterprises’ participation in SWG.
	Constraint level of SWG (X9)	Take environmental protection tax as government constraint level on enterprise behaviors in SWG.
Output variable (Y)	Water consumption of GDP (Y1)	It means the amount of water consumed per 10,000 yuan of GDP.
	Water consumption of industrial added value (Y2)	It means the amount of water consumed per 10,000 yuan increase in industrial production value.
	Daily sewage treatment capacity (Y3)	It represents the total daily sewage treatment.
Environment variable (Z)	Public population pressure (Z1)	Take population density as public population pressure.
	Public consumption capacity (Z2)	Take GDP per capita as the public consumption capacity.
	Per capita water resources (Z3)	It means the per capita water holdings.
	Public knowledge level (Z4)	Take the number of higher education students per 100,000 people as the public knowledge level.
	Urbanization level (Z5)	It represents the ratio of the urban area to the total area.

**Table 2:** Descriptive statistics of SWCG indexes.

Index Type	Index	Method of Calculation	Max	Min	Mean	S.D.
Input variable (X)	X1	Government expenditure on science and technology (10^9^ Yuan)	1168.79	7.28	192.08	239.74
	X2	Government expenditure on energy conservation and environmental protection (10^9^ Yuan)	747.44	40.91	224.81	142.37
	X3	Number of employees in scientific research and technology (10^5^)	68.90	1.20	14.01	14.21
	X4	Number of employees in the water conservancy environment (10^5^)	18.00	0.50	7.89	4.37
	X5	Length of water supply pipe/built-up area (km·km^−2^)	31.40	7.22	12.26	4.77
	X6	Length of water drainage pipe/built-up area (km·km^−2^)	19.17	5.08	9.83	3.58
	X7	Regional water consumption/total water resources (%)	5.55	0.01	0.82	1.22
	X8	Number of enterprises with pollutant discharge permits	37,983.00	525.00	10,244.77	9164.51
	X9	Environmental protection tax (10^9^ Yuan)	35.89	0.20	7.13	8.00
Output variable (Y)	Y1	Water consumption/GDP (m^3^·10^5^ Yuan^−1^)	984.05	11.80	129.23	131.45
	Y2	Water consumption/industrial value added (m^3^·10^5^ Yuan^−1^)	377.55	7.39	60.55	49.41
	Y3	Total sewage treatment capacity/365 (10^5^ ton·day^−1^)	2453.10	4.20	507.65	446.32
Environment variable (Z)	Z1	Urban population/urban area (people·km^−2^)	5498.00	1137.00	3008.10	1121.61
	Z2	GDP/total population (10^5^ Yuan)	164,220.00	32,995.00	69,235.06	32,698.43
	Z3	Total water resources/total population (m^3^·people^−1^)	129,407.20	51.90	6303.13	23,016.58
	Z4	Higher education students/total population (per 10^6^ people)	5320.00	1486.00	2833.06	741.08
	Z5	Urban area/total area (%)	0.88	0.32	0.61	0.12

**Table 3:** Top ten provinces regarding the 2019 China Internet Development Index.

Type	Province
Top ten provinces in the 2019 China Internet Development Index	Beijing, Guangdong, Shanghai, Jiangsu, Zhejiang, Shandong, Sichuan, Fujian, Tianjin, Chongqing
Others (Excluding Hong Kong, Macao, and Taiwan)	Hebei, Shanxi, Inner Mongolia, Liaoning, Jilin, Heilongjiang, Anhui, Jiangxi, Henan, Hubei, Hunan, Guangxi, Hainan, Guizhou, Yunnan, Tibet, Shaanxi, Gansu, Qinghai, Ningxia, Xinjiang

**Table 4:** SFA results of input and environment variables.

Variable	Slack Variable								
	X1	X2	X3	X4	X5	X6	X7	X8	X9
Constant	−100.58 *** (−37.12)	−172.02 *** (−168.31)	−18.11 *** (−11.49)	−18.11 (0.50)	0.27 (0.40)	0.05 (0.27)	−0.87 (−1.56)	−741.92 *** (−741.92)	−7.23 *** (−7.22)
Z1	−0.01 (−0.82)	0.00 *** (4.27)	−0.01 (−1.39)	−0.01 (−0.03)	0.00 (0.38)	0.00 (0.36)	0.00 (1.33)	0.26 *** (9.37)	0.00 (−1.28)
Z2	3.18 ** (3.57)	−9.69 *** (−12.82)	0.42 (0.86)	0.42 (0.17)	0.01 (0.08)	0.01 (0.59)	−0.03 (−1.37)	−152.29 *** (−152.29)	−0.80 *** (−6.62)
Z3	0.00 *** (30.03)	0.00 (2.00)	0.00 *** (5.38)	0.00 (−0.54)	0.00 (−1.12)	0.00 (−0.36)	0.00 (0.92)	0.01 (0.15)	0.00 (0.57)
Z4	38.65 *** (19.51)	11.74 *** (9.05)	7.59 *** (6.60)	7.59 (0.04)	−0.33 * (−2.47)	−0.01 (−0.11)	0.09 (1.60)	−387.43 *** (−387.43)	−1.46 * (−2.52)
Z5	−82.09 *** (−77.18)	276.96 *** (271.23)	−13.44 *** (−11.73)	−13.44 (−1.13)	0.68 (0.38)	−0.18 (−0.40)	1.04 (1.45)	1326.07 *** (1326.07)	24.59 *** (25.80)
*σ* ^2^	5733.74 *** (5734.07)	14,364.45 *** (14,364.43)	97.90 *** (97.50)	97.90 *** (5.11)	5.15 ** (3.00)	10.89 *** (54.99)	0.40 ** (3.68)	1.78 × 10^7^ *** (1.78 × 10^7^)	45.59 *** (43.84)
*γ*	1.00 *** (1.49 × 10^7^)	1.00 *** (6.14 × 10^6^)	1.00 *** (9.42 × 10^4^)	1.00 *** (7760.68)	1.00 *** (1.71 × 10^5^)	1.00 *** (2.01 × 10^7^)	1.00 *** (4.17 × 10^6^)	1.00 *** (12,388.50)	1.00 *** (837.69)
*Log*	−153.99	−168.42	−93.89	−22.32	−46.70	−58.12	−8.38	−277.44	−78.24
*LR*	19.50 ***	19.09 ***	14.01 **	26.20 ***	22.53 ***	24.12 ***	23.15 ***	21.77 ***	21.27 ***

Note: *LR* is a likelihood ratio test, subject to a mixed chi-square distribution. *, **, and *** indicate significance at the 10%, 5%, and 1% levels, respectively. In this test, the critical *LR* values at the 1%, 5%, and 10% significance levels were 8.57, 10.37, and 14.33, respectively.

**Table 5:** The ratio of redundant investment and inefficient production in non-DEA-efficiency provinces (%).

Province	Output Variables			Input Variables								
	Y1	Y2	Y3	X1	X2	X3	X4	X5	X6	X7	X8	X9
Beijing	0.34	0.34	0.34	0.92	0.57	3.01	0.04	0.00	0.05	0.85	0.00	0.00
Tianjin	0.39	0.39	0.39	1.78	1.94	1.16	0.00	0.96	2.24	13.56	0.00	0.49
Hebei	0.42	0.42	0.42	0.00	2.68	0.60	0.21	0.01	0.51	1.68	1.34	2.94
Shanxi	1.32	1.32	1.32	0.00	1.13	0.01	0.38	0.11	0.65	1.04	0.24	2.19
Inner Mongolia	0.31	0.31	0.31	0.00	1.23	0.78	0.45	0.35	0.28	1.54	0.68	7.15
Liaoning	0.00	0.00	0.00	0.00	0.92	0.01	0.30	0.10	0.70	0.78	0.18	1.66
Anhui	0.16	0.16	0.16	1.14	1.00	0.00	0.07	0.05	0.10	3.11	0.32	0.00
Fujian	0.53	0.53	0.53	0.05	0.56	0.00	0.02	0.20	0.19	0.19	0.42	0.00
Jiangxi	0.50	0.50	0.50	2.16	1.31	0.15	0.00	0.30	0.24	0.00	1.24	0.09
Henan	0.00	0.00	0.00	0.00	0.00	0.00	0.00	0.00	0.00	0.00	0.00	0.00
Hubei	0.21	0.21	0.21	0.08	0.17	0.13	0.19	0.08	0.00	0.43	0.00	0.11
Hunan	0.11	0.11	0.11	0.00	0.56	0.30	0.35	0.26	0.00	0.00	0.26	0.18
Hainan	0.00	0.00	0.00	0.00	0.00	0.00	0.00	0.00	0.00	0.00	0.00	0.00
Chongqing	0.18	0.18	0.18	0.00	0.74	0.25	0.00	0.35	1.25	0.00	0.22	0.18
Guizhou	0.00	0.00	0.00	0.00	0.00	0.00	0.00	0.00	0.00	0.00	0.00	0.00
Yunnan	0.86	0.86	0.86	0.00	1.30	0.55	0.06	0.17	0.24	0.00	0.31	0.63
Shaanxi	0.00	0.00	0.00	0.00	0.00	0.00	0.00	0.00	0.00	0.00	0.00	0.00
Gansu	0.72	0.72	0.72	0.00	0.12	0.40	0.69	0.00	0.37	0.49	0.46	0.00
Qinghai	0.16	0.16	0.16	0.00	0.55	0.14	0.22	0.36	0.38	0.00	0.44	0.88
Mean	0.20	0.20	0.20	0.20	0.48	0.24	0.10	0.11	0.23	0.76	0.20	0.53

Note: Redundant invest ratio = redundant input value/original input value; inefficient production ratio = inefficient output value/original output value.

## Data Availability

The data of this paper were derived from China Statistical Yearbook 2020. The link is http://www.stats.gov.cn/tjsj/ndsj/2020/indexch.htm (accessed on 20 June 2021). If you are interested in this study and want to obtain the original data, you can contact us.

## Appendix A

A visualization results of the technical and scale efficiency of SWCG in the first and third stages of Section 3.2. are shown in Figure A1.

## Appendix B

The specific numerical results of the first and third stages of SWCG efficiency in Section 3.2. are shown in Table A1.

**Table A1:** Specific numerical results of different stage co-governance efficiency.

Province	Efficiency of G–E–P					Efficiency of G–E				
	Technical	Scale	Co-Governance	Rank	Return to Scale	Technical	Scale	Co-Governance	Rank	Return to Scale
Beijing	0.74	1.00	0.74	22	irs	0.65	0.97	0.62	25	irs
Tianjin	0.72	0.97	0.70	25	irs	0.80	0.81	0.64	22	irs
Hebei	0.70	0.98	0.69	26	drs	0.83	0.81	0.67	20	irs
Shanxi	0.43	0.98	0.42	31	drs	0.72	0.52	0.37	31	irs
Inner Mongolia	0.76	1.00	0.76	21	-	0.77	0.74	0.57	27	irs
Liaoning	1.00	1.00	1.00	1	-	1.00	1.00	1.00	1	-
Jilin	1.00	1.00	1.00	1	-	0.97	0.89	0.86	13	irs
Heilongjiang	1.00	1.00	1.00	1	-	1.00	1.00	1.00	1	-
Shanghai	1.00	1.00	1.00	1	-	1.00	1.00	1.00	1	-
Jiangsu	1.00	1.00	1.00	1	-	1.00	1.00	1.00	1	-
Zhejiang	1.00	1.00	1.00	1	-	1.00	0.93	0.93	11	irs
Anhui	0.86	0.99	0.86	17	drs	0.83	0.94	0.78	18	irs
Fujian	0.66	0.99	0.65	27	irs	0.79	0.70	0.55	28	irs
Jiangxi	0.67	0.95	0.63	28	drs	0.85	0.75	0.63	24	irs
Shandong	1.00	1.00	1.00	1	-	1.00	1.00	1.00	1	-
Henan	1.00	0.93	0.93	15	irs	1.00	0.86	0.86	14	irs
Hubei	0.83	0.97	0.80	20	drs	0.87	0.95	0.82	15	irs
Hunan	0.90	0.98	0.88	16	drs	0.90	0.97	0.87	12	irs
Guangdong	1.00	1.00	1.00	1	-	1.00	1.00	1.00	1	-
Guangxi	1.00	1.00	1.00	1	-	1.00	1.00	1.00	1	-
Hainan	1.00	0.96	0.96	14	irs	1.00	0.60	0.60	26	irs
Chongqing	0.85	0.99	0.84	18	irs	0.97	0.81	0.79	17	irs
Sichuan	1.00	1.00	1.00	1	-	0.95	0.85	0.81	16	irs
Guizhou	1.00	0.83	0.83	19	irs	1.00	0.69	0.69	19	irs
Yunnan	0.54	0.99	0.54	30	irs	0.74	0.64	0.48	30	irs
Tibet	1.00	1.00	1.00	1	-	1.00	1.00	1.00	1	-
Shaanxi	1.00	0.72	0.72	23	irs	1.00	0.65	0.65	21	irs
Gansu	0.58	0.98	0.57	29	irs	0.89	0.60	0.53	29	irs
Qinghai	0.86	0.83	0.72	23	irs	1.00	0.64	0.64	22	irs
Ningxia	1.00	1.00	1.00	1	-	1.00	0.95	0.95	10	irs
Xinjiang	1.00	1.00	1.00	1	-	1.00	1.00	1.00	1	-
Average	0.87	0.97	0.85			0.92	0.85	0.79		

Note: Rank represents the ranking of co-governance efficiency from high to low. “irs” means increasing returns to scale; “drs” means decreasing returns to scale; “-” means constant returns to scale.

The results of the mixed error term in Section 3.3. are shown in Table A2.

**Table A2:** The calculation results of mixed error.

Index	*σ_μ_*	*σ_v_*	*σ **	*λ*
X1	75.72	7.57 × 10^−3^	7.57 × 10^−3^	10,000.00
X2	119.85	1.20 × 10^−2^	1.20 × 10^−2^	10,000.00
X3	9.89	9.89 × 10^−4^	9.89 × 10^−4^	10,000.00
X4	1.20	1.20 × 10^−4^	1.20 × 10^−4^	10,000.00
X5	2.27	2.27 × 10^−4^	2.27 × 10^−4^	10,000.00
X6	3.30	3.30 × 10^−4^	3.30 × 10^−4^	10,000.00
X7	0.63	6.29 × 10^−5^	6.29 × 10^−5^	10,000.00
X8	4214.30	4.21 × 10^−1^	4.21 × 10^−1^	10,000.00
X9	6.75	6.75 × 10^−4^	6.75 × 10^−4^	10,000.00

The complete calculation results of the Malmquist index in 31 provinces from 2010 to 2019 in Section 3.4.1. are shown in Table A3.

**Table A3:** 2010–2019 Malmquist complete results in 31 provinces, China.

Province	2010–2011					2011–2012					2012–2013				
	EFF	TECH	PE	SE	TFP	EFF	TECH	PE	SE	TFP	EFF	TECH	PE	SE	TFP
Beijing	1.00	0.92	1.00	1.00	0.92	1.00	1.10	1.00	1.00	1.10	1.00	0.83	1.00	1.00	0.83
Tianjin	1.36	0.72	1.06	1.28	0.98	1.03	0.98	1.02	1.00	1.00	0.97	0.88	0.87	1.12	0.86
Hebei	1.48	0.69	1.00	1.48	1.02	0.96	0.94	1.00	0.96	0.91	0.84	1.20	0.88	0.96	1.01
Shanxi	1.50	0.70	1.08	1.39	1.04	0.95	0.94	0.91	1.05	0.90	0.88	0.84	0.54	1.64	0.74
Inner Mongolia	1.21	0.75	0.99	1.23	0.91	1.11	0.86	0.88	1.26	0.96	0.97	0.87	0.67	1.45	0.84
Liaoning	1.52	0.71	1.16	1.31	1.07	0.91	1.01	0.91	1.01	0.92	1.31	0.91	1.15	1.14	1.19
Jilin	1.26	0.73	1.00	1.26	0.92	1.03	0.96	1.00	1.03	0.98	0.78	1.01	0.59	1.31	0.78
Heilongjiang	1.12	0.82	1.00	1.12	0.91	0.89	0.99	1.00	0.89	0.88	1.13	1.23	1.00	1.13	1.39
Shanghai	1.02	0.94	1.00	1.02	0.96	1.00	1.07	1.00	1.00	1.07	1.00	0.92	1.00	1.00	0.92
Jiangsu	1.02	0.87	1.00	1.02	0.89	1.00	0.93	1.00	1.00	0.93	1.00	0.85	1.00	1.00	0.85
Zhejiang	1.15	0.67	1.11	1.04	0.77	1.25	1.10	1.27	0.98	1.38	1.04	0.85	1.07	0.98	0.89
Anhui	0.92	0.84	0.96	0.96	0.78	0.90	1.03	0.89	1.01	0.93	1.31	0.85	1.15	1.14	1.12
Fujian	1.48	0.69	1.22	1.22	1.02	1.12	0.93	1.06	1.05	1.04	1.15	0.88	1.06	1.08	1.01
Jiangxi	1.18	0.74	1.07	1.10	0.87	0.99	0.91	0.86	1.16	0.90	1.07	0.87	0.89	1.21	0.93
Shandong	1.18	0.87	1.00	1.18	1.03	0.99	0.96	1.00	0.99	0.95	1.03	0.95	1.00	1.03	0.98
Henan	1.22	0.77	1.00	1.22	0.95	1.02	0.96	1.02	1.00	0.98	1.23	1.02	1.01	1.21	1.26
Hubei	1.29	0.73	1.13	1.14	0.94	0.92	0.98	0.94	0.99	0.91	1.15	1.00	1.19	0.97	1.14
Hunan	1.19	0.74	1.07	1.11	0.89	1.01	0.97	1.00	1.01	0.98	0.81	1.04	0.77	1.05	0.85
Guangdong	1.00	0.80	1.00	1.00	0.80	1.00	1.13	1.00	1.00	1.13	1.00	1.01	1.00	1.00	1.01
Guangxi	1.00	0.69	1.00	1.00	0.69	1.00	0.91	1.00	1.00	0.91	1.00	0.89	1.00	1.00	0.89
Hainan	1.17	0.95	1.01	1.16	1.11	1.00	0.95	1.00	1.00	0.94	1.03	0.87	1.00	1.03	0.89
Chongqing	1.47	0.75	1.22	1.21	1.11	0.92	1.00	0.90	1.02	0.91	1.11	0.85	0.78	1.42	0.95
Sichuan	1.41	0.64	1.15	1.23	0.90	0.86	1.16	0.86	1.00	1.00	1.12	0.87	1.09	1.03	0.97
Guizhou	1.01	0.78	0.96	1.05	0.79	0.91	1.10	0.92	0.99	1.00	1.23	1.04	1.12	1.09	1.27
Yunnan	1.20	0.69	0.98	1.22	0.83	0.90	1.09	0.90	1.00	0.98	1.03	0.88	0.71	1.46	0.91
Tibet	1.00	0.73	1.00	1.00	0.73	1.00	0.78	1.00	1.00	0.78	1.00	1.14	1.00	1.00	1.14
Shaanxi	1.26	0.74	1.00	1.26	0.94	1.02	0.95	1.00	1.02	0.97	0.93	1.21	0.81	1.14	1.12
Gansu	1.17	0.77	1.00	1.17	0.90	1.16	0.86	1.00	1.16	1.00	0.74	1.11	0.63	1.17	0.82
Qinghai	1.97	0.59	1.36	1.45	1.17	0.80	0.67	0.91	0.88	0.54	1.04	0.91	0.74	1.40	0.95
Ningxia	1.18	0.76	1.00	1.18	0.89	1.00	0.89	1.00	1.00	0.89	1.00	0.84	1.00	1.00	0.84
Xinjiang	1.00	0.84	1.00	1.00	0.84	1.00	0.91	1.00	1.00	0.91	0.95	0.87	1.00	0.95	0.83
Mean	1.21	0.76	1.05	1.15	0.91	0.99	0.96	0.97	1.01	0.95	1.02	0.95	0.91	1.12	0.96
**Province**	**2013–2014**					**2014–2015**					**2015–2016**				
	**EFF**	**TECH**	**PE**	**SE**	**TFP**	**EFF**	**TECH**	**PE**	**SE**	**TFP**	**EFF**	**TECH**	**PE**	**SE**	**TFP**
Beijing	0.60	0.61	0.64	0.95	0.37	0.98	1.02	0.98	1.00	1.00	0.97	0.92	0.93	1.05	0.90
Tianjin	0.93	0.99	1.05	0.89	0.92	1.05	0.96	1.10	0.95	1.01	0.97	1.05	0.94	1.04	1.03
Hebei	0.95	1.02	0.97	0.97	0.97	1.10	1.01	1.07	1.03	1.11	0.89	1.02	0.91	0.97	0.90
Shanxi	1.15	0.97	1.11	1.04	1.11	1.12	1.07	1.24	0.90	1.20	1.02	1.10	1.05	0.97	1.12
Inner Mongolia	1.09	0.98	1.04	1.05	1.07	1.02	1.06	1.04	0.98	1.08	1.11	1.08	1.22	0.91	1.19
Liaoning	0.98	0.98	1.00	0.98	0.97	1.02	1.00	1.00	1.02	1.02	1.00	1.17	1.00	1.00	1.17
Jilin	1.03	0.99	1.03	1.01	1.02	1.20	1.07	1.21	0.98	1.28	0.89	1.05	0.99	0.90	0.94
Heilongjiang	1.00	0.99	1.00	1.00	0.99	1.00	0.95	1.00	1.00	0.95	1.00	1.02	1.00	1.00	1.02
Shanghai	1.00	0.98	1.00	1.00	0.98	1.00	0.85	1.00	1.00	0.85	0.93	0.88	0.95	0.98	0.82
Jiangsu	1.00	0.96	1.00	1.00	0.96	1.00	0.98	1.00	1.00	0.98	1.00	1.03	1.00	1.00	1.03
Zhejiang	0.97	0.96	0.94	1.04	0.93	0.99	0.99	1.00	0.99	0.97	1.01	1.06	0.99	1.03	1.07
Anhui	0.94	0.98	0.94	1.00	0.93	1.06	1.00	1.05	1.01	1.06	0.75	1.08	0.78	0.95	0.81
Fujian	0.95	0.95	1.00	0.95	0.90	0.83	0.90	0.88	0.95	0.75	0.92	1.09	0.83	1.11	1.00
Jiangxi	1.10	0.97	1.10	1.00	1.07	1.03	0.96	1.03	1.01	0.99	0.80	1.09	0.92	0.87	0.87
Shandong	1.00	1.13	1.00	1.00	1.13	1.00	0.98	1.00	1.00	0.98	1.00	1.06	1.00	1.00	1.06
Henan	0.92	1.10	1.00	0.92	1.01	1.11	0.97	1.00	1.11	1.07	0.96	1.02	1.00	0.96	0.98
Hubei	0.93	1.04	0.97	0.95	0.97	1.05	0.84	1.02	1.03	0.88	1.05	0.92	1.07	0.99	0.97
Hunan	1.10	1.00	1.09	1.01	1.10	1.09	1.03	1.08	1.00	1.12	0.96	1.09	0.98	0.99	1.05
Guangdong	1.00	1.00	1.00	1.00	1.00	1.00	0.91	1.00	1.00	0.91	1.00	1.07	1.00	1.00	1.07
Guangxi	1.00	0.98	1.00	1.00	0.98	1.00	1.04	1.00	1.00	1.04	1.00	0.88	1.00	1.00	0.88
Hainan	0.97	1.03	1.00	0.97	0.99	1.15	0.72	1.00	1.15	0.83	0.79	0.96	1.00	0.79	0.77
Chongqing	0.94	1.01	0.94	1.01	0.95	0.88	1.02	0.87	1.01	0.90	0.90	1.14	0.99	0.91	1.02
Sichuan	1.10	1.06	1.09	1.00	1.16	0.85	1.00	0.89	0.95	0.85	1.13	0.98	1.15	0.99	1.10
Guizhou	0.69	1.02	0.91	0.75	0.70	1.08	0.80	1.12	0.96	0.86	1.27	0.82	1.30	0.97	1.04
Yunnan	0.97	1.02	0.97	1.00	0.99	0.95	1.16	0.96	0.99	1.10	1.15	0.92	1.11	1.04	1.06
Tibet	1.00	0.79	1.00	1.00	0.79	1.00	0.63	1.00	1.00	0.63	1.00	0.95	1.00	1.00	0.95
Shaanxi	1.04	1.02	1.01	1.03	1.07	1.19	0.92	1.05	1.14	1.10	0.93	1.04	1.12	0.83	0.97
Gansu	0.92	1.02	1.00	0.92	0.94	0.94	0.83	1.07	0.89	0.79	1.03	0.88	1.15	0.89	0.90
Qinghai	0.92	1.03	0.95	0.97	0.95	1.22	0.95	1.15	1.06	1.15	0.90	1.27	0.95	0.95	1.14
Ningxia	1.00	0.82	1.00	1.00	0.82	1.00	0.94	1.00	1.00	0.94	1.00	0.98	1.00	1.00	0.98
Xinjiang	1.05	0.85	1.00	1.05	0.89	1.00	0.95	1.00	1.00	0.95	1.00	0.97	1.00	1.00	0.97
Mean	0.97	0.97	0.99	0.98	0.94	1.02	0.95	1.02	1.00	0.97	0.97	1.01	1.01	0.97	0.99
**Province**	**2016–2017**					**2017–2018**					**2018–2019**				
	**EFF**	**TECH**	**PE**	**SE**	**TFP**	**EFF**	**TECH**	**PE**	**SE**	**TFP**	**EFF**	**TECH**	**PE**	**SE**	**TFP**
Beijing	0.98	1.01	0.98	1.00	0.99	0.86	1.12	0.89	0.97	0.96	0.96	0.93	1.25	0.77	0.90
Tianjin	1.00	0.98	0.82	1.22	0.98	1.05	1.15	1.26	0.84	1.21	0.99	0.99	0.81	1.22	0.98
Hebei	1.00	0.95	1.02	0.99	0.95	0.82	1.21	0.90	0.92	1.00	1.13	0.91	1.08	1.05	1.03
Shanxi	0.94	0.93	0.94	1.00	0.87	0.84	1.16	1.00	0.83	0.97	1.16	0.93	1.14	1.02	1.08
Inner Mongolia	1.14	0.89	1.02	1.12	1.01	1.27	0.97	1.15	1.10	1.23	0.97	0.95	0.94	1.04	0.93
Liaoning	1.00	0.91	1.00	1.00	0.91	1.00	1.27	1.00	1.00	1.27	1.00	0.89	1.00	1.00	0.89
Jilin	1.39	0.88	1.25	1.11	1.22	1.29	1.27	1.09	1.19	1.63	1.00	0.92	1.00	1.00	0.92
Heilongjiang	1.00	0.70	1.00	1.00	0.70	1.00	1.16	1.00	1.00	1.16	1.00	1.00	1.00	1.00	1.00
Shanghai	0.82	0.97	0.82	1.00	0.80	1.31	1.28	1.28	1.02	1.68	1.00	0.85	1.00	1.00	0.85
Jiangsu	1.00	1.05	1.00	1.00	1.05	1.00	1.11	1.00	1.00	1.11	1.00	0.91	1.00	1.00	0.91
Zhejiang	0.98	0.95	1.00	0.99	0.94	1.12	1.26	1.10	1.02	1.40	1.00	0.89	1.00	1.00	0.89
Anhui	1.07	1.02	1.03	1.04	1.08	1.11	1.11	1.09	1.02	1.23	1.01	0.91	1.02	0.99	0.91
Fujian	1.01	1.01	1.01	1.00	1.01	0.89	1.17	0.91	0.97	1.04	1.00	0.89	1.03	0.98	0.89
Jiangxi	1.01	1.03	0.88	1.15	1.04	0.89	1.17	0.95	0.94	1.04	1.25	0.88	1.14	1.10	1.11
Shandong	1.00	1.03	1.00	1.00	1.03	1.00	1.04	1.00	1.00	1.04	1.00	0.94	1.00	1.00	0.94
Henan	1.04	0.99	1.00	1.04	1.03	0.90	1.05	1.00	0.90	0.94	1.03	0.93	1.00	1.03	0.95
Hubei	0.95	0.96	0.95	1.01	0.92	0.89	1.02	0.81	1.09	0.91	1.01	0.88	1.06	0.96	0.89
Hunan	1.03	0.93	1.06	0.97	0.95	0.96	1.06	0.91	1.05	1.02	1.04	0.92	1.06	0.98	0.96
Guangdong	1.00	1.00	1.00	1.00	1.00	1.00	1.18	1.00	1.00	1.18	1.00	0.98	1.00	1.00	0.98
Guangxi	1.00	0.88	1.00	1.00	0.88	1.00	1.08	1.00	1.00	1.08	1.00	0.88	1.00	1.00	0.88
Hainan	1.29	1.04	1.00	1.29	1.35	0.76	1.17	1.00	0.76	0.89	1.14	0.80	1.00	1.14	0.91
Chongqing	1.04	1.00	1.16	0.90	1.05	1.15	1.14	1.05	1.10	1.32	1.39	0.88	1.25	1.12	1.23
Sichuan	1.15	0.94	1.04	1.11	1.09	1.00	1.04	1.00	1.00	1.04	1.00	1.01	1.00	1.00	1.01
Guizhou	1.59	0.85	1.12	1.43	1.35	0.89	0.90	1.00	0.89	0.80	1.12	0.88	1.00	1.12	0.99
Yunnan	1.01	0.99	1.01	1.00	1.00	1.05	0.94	1.04	1.01	0.98	0.83	1.04	1.03	0.81	0.87
Tibet	1.00	0.73	1.00	1.00	0.73	1.00	0.65	1.00	1.00	0.65	1.00	0.77	1.00	1.00	0.77
Shaanxi	0.98	0.93	0.96	1.02	0.92	1.08	1.13	1.07	1.00	1.22	1.19	0.90	1.00	1.19	1.07
Gansu	1.23	0.87	1.01	1.22	1.07	0.96	1.02	0.94	1.02	0.98	0.99	0.91	1.16	0.85	0.90
Qinghai	1.02	0.79	1.16	0.88	0.81	0.96	1.00	0.95	1.00	0.95	1.47	0.96	1.20	1.23	1.41
Ningxia	1.00	0.98	1.00	1.00	0.98	1.00	1.20	1.00	1.00	1.20	1.00	0.96	1.00	1.00	0.96
Xinjiang	1.00	1.00	1.00	1.00	1.00	1.00	0.76	1.00	1.00	0.76	1.00	0.94	1.00	1.00	0.94
Mean	1.05	0.94	1.00	1.04	0.98	0.99	1.08	1.01	0.99	1.07	1.05	0.92	1.03	1.01	0.96

The mean results of Malmquist index in 31 provinces from 2010 to 2019 in Section 3.4.2. are shown in Table A4.

**Table A4:** 2010–2019 Malmquist mean results in 31 provinces, China.

Province	EFFCH	TECHCH	PECH	SECH	TFPCH	Province	EFFCH	TECHCH	PECH	SECH	TFPCH
Beijing	0.92	0.93	0.95	0.97	0.85	Hubei	1.02	0.93	1.01	1.01	0.94
Tianjin	1.03	0.96	0.98	1.05	0.99	Hunan	1.02	0.97	1.00	1.02	0.99
Hebei	1.00	0.98	0.98	1.03	0.99	Guangdong	1.00	1.00	1.00	1.00	1.00
Shanxi	1.05	0.95	0.98	1.07	0.99	Guangxi	1.00	0.91	1.00	1.00	0.91
Inner Mongolia	1.09	0.93	0.98	1.12	1.02	Hainan	1.02	0.93	1.00	1.02	0.95
Liaoning	1.07	0.97	1.02	1.05	1.04	Chongqing	1.07	0.97	1.01	1.07	1.04
Jilin	1.08	0.98	1.00	1.08	1.05	Sichuan	1.06	0.95	1.03	1.03	1.01
Heilongjiang	1.01	0.97	1.00	1.01	0.99	Guizhou	1.06	0.90	1.04	1.01	0.96
Shanghai	1.00	0.96	1.00	1.00	0.97	Yunnan	1.00	0.96	0.96	1.05	0.96
Jiangsu	1.00	0.96	1.00	1.00	0.96	Tibet	1.00	0.78	1.00	1.00	0.78
Zhejiang	1.05	0.96	1.05	1.01	1.01	Shaanxi	1.06	0.98	1.00	1.06	1.04
Anhui	1.00	0.98	0.99	1.01	0.97	Gansu	1.00	0.91	0.98	1.02	0.92
Fujian	1.02	0.94	0.99	1.03	0.96	Qinghai	1.10	0.89	1.03	1.07	0.98
Jiangxi	1.03	0.95	0.98	1.05	0.98	Ningxia	1.02	0.92	1.00	1.02	0.94
Shandong	1.02	0.99	1.00	1.02	1.01	Xinjiang	1.00	0.90	1.00	1.00	0.90
Henan	1.04	0.97	1.00	1.04	1.01	Average	1.03	0.94	1.00	1.03	0.97
